# Characterization of a pneumococcal meningitis mouse model

**DOI:** 10.1186/1471-2334-12-71

**Published:** 2012-03-28

**Authors:** Barry Mook-Kanamori, Madelijn Geldhoff, Dirk Troost, Tom van der Poll, Diederik van de Beek

**Affiliations:** 1 Department of Neurology, Center of Infection and Immunity Amsterdam (CINIMA), Academic Medical Center, Amsterdam, the Netherlands; 2 Department of (Neuro) Pathology, Academic Medical Center, Amsterdam, the Netherlands; 3 Center for Experimental and Molecular Medicine (CEMM), Center of Infection and Immunity Amsterdam (CINIMA), Academic Medical Center, Amsterdam, the Netherlands; 4 Department of Neurology, Center of Infection and Immunity Amsterdam (CINIMA), Academic Medical Center, University of Amsterdam, P.O. Box 22660, Amsterdam 1100DD, The Netherlands

**Keywords:** Meningitis, Critical care, Neurology, Animal model, Infectious diseases

## Abstract

**Background:**

*S. pneumoniae *is the most common causative agent of meningitis, and is associated with high morbidity and mortality. We aimed to develop an integrated and representative pneumococcal meningitis mouse model resembling the human situation.

**Methods:**

Adult mice (C57BL/6) were inoculated in the cisterna magna with increasing doses of *S. pneumoniae *serotype 3 colony forming units (CFU; n = 24, 10^4^, 10^5^, 10^6 ^and 10^7 ^CFU) and survival studies were performed. Cerebrospinal fluid (CSF), brain, blood, spleen, and lungs were collected. Subsequently, mice were inoculated with 10^4 ^CFU *S. pneumoniae *serotype 3 and sacrificed at 6 (n = 6) and 30 hours (n = 6). Outcome parameters were bacterial outgrowth, clinical score, and cytokine and chemokine levels (using Luminex^®^) in CSF, blood and brain. Meningeal inflammation, neutrophil infiltration, parenchymal and subarachnoidal hemorrhages, microglial activation and hippocampal apoptosis were assessed in histopathological studies.

**Results:**

Lower doses of bacteria delayed onset of illness and time of death (median survival CFU 10^4^, 56 hrs; 10^5^, 38 hrs, 10^6^, 28 hrs. 10^7^, 24 hrs). Bacterial titers in brain and CSF were similar in all mice at the end-stage of disease independent of inoculation dose, though bacterial outgrowth in the systemic compartment was less at lower inoculation doses. At 30 hours after inoculation with 10^4 ^CFU of *S. pneumoniae*, blood levels of KC, IL6, MIP-2 and IFN- γ were elevated, as were brain homogenate levels of KC, MIP-2, IL-6, IL-1β and RANTES. Brain histology uniformly showed meningeal inflammation at 6 hours, and, neutrophil infiltration, microglial activation, and hippocampal apoptosis at 30 hours. Parenchymal and subarachnoidal and cortical hemorrhages were seen in 5 of 6 and 3 of 6 mice at 6 and 30 hours, respectively.

**Conclusion:**

We have developed and validated a murine model of pneumococcal meningitis.

## Background

Bacterial meningitis is a life threatening infectious disease of the central nervous system (CNS). The annual incidence is estimated to be up to 2.6 to 6.0 cases per 100 000 in Europe and may be up to ten times higher in developing countries [1,2]. The most common pathogen beyond the neonatal period is *Streptococcus pneumoniae *[1,3], causing 70% of cases. Despite advances in medical care, mortality from pneumococcal meningitis remains between 16% and 37% and neurological sequelae affect 30-52% of survivors [4-6]. There is a continuing need for the development of new treatment strategies.

Complications associated with pneumococcal meningitis include cerebral infarction, hemorrhages, motor and sensory deficit, seizures, memory and learning impairments, and hearing loss [2,7,8]. Autopsy studies of patients who died following pneumococcal meningitis revealed cerebral edema, cerebral infarctions and hemorrhages, apoptosis and necrosis of the hippocampal dentate gyrus [9-11]. Many of these pathological features have been reproduced in animal models, which provide the setting for novel drug development and pathophysiological studies [12,13].

Several murine models have been developed, using intracerebral [14,15], intraperitoneal [16], intravenous [16], intranasal [17] or intracisternal inoculation methods [18,19], and have recently been reviewed [12]. Problems with reproducibility, limited disease progression or iatrogenic structural damage, combined with a need for a single model in which most pathological features seen in human pneumococcal meningitis can be measured, has fueled the development of new animal models. Here we describe the development of an adult mouse model of pneumococcal meningitis in which many of the human pathological features are demonstrated.

## Methods

A clinical isolate of *S. pneumoniae *serotype 3 was obtained from ATCC (catalog number 6303), and was grown to mid log phase in 4 hours at 37°C in Todd-Hewitt broth supplemented with 0.5% yeast extract. At an OD_620 _of 0.8 to 1.0 the *S. pneumoniae *were centrifuged and washed twice by resuspension in sterile 0.9% NaCl and recentrifugation. Finally, the bacteria were resuspended in sterile NaCl 0.9% to yield an approximate concentration of 1 × 10^9 ^colony forming units (CFU)/ml. The exact number of CFUs was subsequently determined for inoculates by serial dilution method and on blood agar plates (overnight at 37°C).

Animal experiments were approved by the Institutional Animal Care and Use Committee of the Academic Medical Center, Amsterdam. To determine the inoculation dose and optimal time points of sacrifice, 24 8-10 week old male C57BL/6 mice (Charles River Laboratories, Germany) received 0.1 mg/kg s.c. buprenorphine and short-term anesthesia using 1.5-2.0% isoflurane during inoculation. The mice were divided into 4 groups, each receiving a different concentration of bacterial inoculum (10^4^, 10^5^, 10^6 ^and 10^7 ^CFU *S. pneumoniae *per mouse; n = 6 per dose). Inoculation was conducted by injecting 10 μL of bacterial suspension into the cisterna magna using a 32-gauge needle. All animals were evaluated directly following inoculation and subsequently at 4-hour intervals. The following scoring was used (Table 1): range: 0-41 pts; each scoring parameter ranging from 0, corresponding to no deficit, to a variable maximum score. The maximum score was determined by the estimated contribution of the variable to overall health of the mouse): weight loss (0-4 pts), activity (0-4 pts), time to return to upright position (0-6 pts), state of skin/fur (0-3 pts), posture (0-2 pts), eye discharge or protrusion (0-4 pts), respiration rate (0-4 pts), irregular/labored breathing (0-4 pts), epilepsy, limb paresis or ataxia (0-10pts). The clinical course was divided into a pre-symptomatic period (from time of inoculation until clinical score ≤ 10) and symptomatic period (clinical score > 10 until death/sacrifice). Survival studies were performed and cerebrospinal fluid (CSF), brain, blood, spleen, and lungs were collected post mortem. After determining inoculation dose and time-points of sacrifice, the model was further characterized using 12 additional mice inoculated with 10^4 ^CFU *S. pneumoniae *serotype 3 and sacrificed at 6 (n = 6) and 30 hours (n = 6).

**Table 1:** Clinical score parameters, assessed values and weighted scores

Parameter	Value	Weighted score	Maximum score
Weight loss from baseline	5%	0	
	10%	1	
	15%	2	
	20%	3	
	25%	4	4

Activity	normal	0	
	increased/decreased	1	
	mildely deminished	1	
	deminished	2	
	severely deminished	3	
	coma	4	4

Time to return to upright	normal	0	
position	upright < 5 sec	2	
	upright < 30 sec	4	
	no turn upright	6	6

Coat	normal	0	
	deminished grooming	1	
	soiled	1	
	piloerection	1	3

Posture	normal	0	
	sligh hunched back	1	
	sev hunched back	2	2

Eyes	normal	0	
	protruding	1	
	sunken eyes	1	
	closed eyelids	1	
	discharge	1	4

Respiration rate (per min)	> 150	0	
	< 150	1	
	< 100	2	
	< 75	3	
	< 50	4	4

Breathing	irregular	2	
	laboured	2	4

Neurologic exam	normal	0	
	ataxia	2	
	limb paresis/paralysis	2	
	epileptic seizure	2	
	status epilepticus	6	10

Total			41

Bacterial titers were determined in samples of lung, brain, and spleen (diluted 1:4 in sterile NaCl 0.9% and homogenized). Blood was heparinized in a 1:4 dilution and CSF was diluted 1:100 in sterile NaCl 0.9%. All bacterial titers were determined by plating serial dilutions on blood agar plates and incubating overnight at 37 C.

Cytokine and chemokine measurement were performed on the left cerebral hemisphere diluted 1:4 in sterile NaCl 0.9%, homogenized and lysed in lysis buffer (150 mM NaCl, 15 mM Tris, 1 mM MgCl(H_2_O)_6_, 1 mM CaCl_2_(H_2_O)_2_, 1% Triton, AEBSF 4 μg/ml, EDTA-NA2 50 μg/ml, pepstatin 10 ng/ml, leupeptin 10 ng/ml, pH 7.4). Samples of brain homogenate, serum and CSF were then centrifuged and supernatant stored at -80 C. Cytokine concentrations were determined with luminex^® ^technology using a mouse cytokine and chemokine Bioplex kit (Bio-Rad Laboratories, Veenendaal, The Netherlands).

All mice were perfused by cardiac puncture with PBS prior to harvesting tissue. Histopathology was performed on the right cerebral hemisphere fixed in 4% paraformaldehyde and paraffin embedded. Coronal 10 μm sections of the entire hemisphere were cut for subsequent staining. Hematoxylin and eosin (HE) and Nissl staining were performed to visualize hemorrhages, cortical necrosis, vasculitis and abscess formation. To determine neuronal apoptosis in the dentate gyrus of the hippocampus, four 10 μm sections of the anterior, middle and posterior portion of the hippocampus were stained with Caspase-3 antibodies (polyclonal rabbit-anti-mouse, 1:100; Cell Signalling, Danvers, MA). In each section, the total number of caspase-3 positive cells was counted in both the dentate gyrus (DG) and cornu ammonis (CA) regions. Scoring was independently conducted by two investigators. Microglial activation was evaluated by immunohistochemistry using Iba-1 antibody (polyclonal rabbit-anti-mouse, 1:2000; ABcam, Cambridge, UK) staining of frontal lobe 10 μm sections. No quantitative analysis was performed.

Comparisons of cytokine levels between groups were calculated using the Mann-Whitney U test. A Kruskal-Wallis one-way ANOVA was used to compare clinical scores of pre-symptomatic and symptomatic periods. Histopathological scores of neuronal apoptosis were compared using Student's *t*-test. For all analyses a p-value < 0.05 was considered significant.

## Results

**Figure 1:**
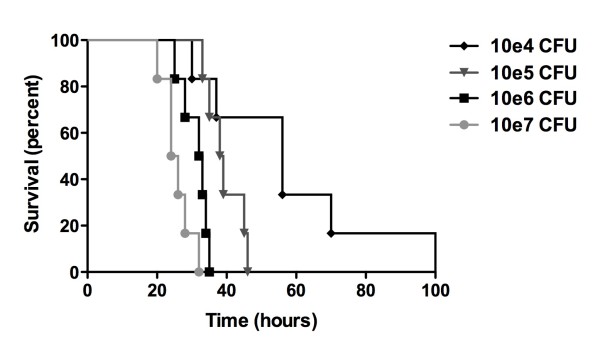
**Kaplan-Meier survival curve**. Four groups of 6 mice inoculated via direct intracisternal injection with 10^4^, 10^5^, 10^6 ^and 10^7 ^CFU of *S. pneumoniae*/mouse respectively.

Mortality occurred in nearly all inoculated mice (Figure 1); one mouse inoculated with 10^4 ^CFU survived beyond the study window of 216 hours after inoculation and was sacrificed. The median survival time was dose dependent (10^4 ^CFU, 56 hrs; 10^5^, 38 hrs, 10^6^:, 28 hrs; 10^7^, 24 hrs). To approximate a physiological setting, we selected 10^4 ^CFU as the lowest concentration of bacterial inoculum in which most animals would die if left untreated. Furthermore, for future experimentation we chose 30 hours post inoculation as the latest time point for sacrifice, at which all animals were still alive and the natural course of the infection could be followed as long as possible.

Clinical scoring was performed on all mice in the survival study. The average duration of the pre-symptomatic period (clinical score ≤ 10) was dose dependent, and increased approximately 1.5-fold with each successive 10-fold increase of bacterial inoculum concentration. The duration of the symptomatic period did not differ significantly between inoculation doses (mean, 11.7 hrs, SD 4.8; Table 2).

**Table 2:** Duration of pre-symptomatic (time from inoculation to clinical score ≤ 10) and symptomatic (time from clinical score > 10 to death/sacrifice) periods

*Inoculation dose (CFU/mouse)*	*10e4*	*10e5*	*10e6*	*10e7*	*p-value*
Pre-symptomatic period (hrs/st.dev)	40.7 (14.9)	26.1 (6.1)	18.7 (3.9)	12.0 (2.1)	0.001

Symptomatic period (hrs/st.dev)	9.1 (3.2)	13.0 (6.3)	10.8 (3.6)	13.6 (5.3)	0.557

Mean durations of the pre-symptomatic and symptomatic periods (expressed in hours) of the mice of the survival study. Mice were divided into 4 groups and inoculated with 104 (5 mice, 1 mouse was excluded from analysis due to lack of any symptoms due to failed inoculation), 105 (6 mice), 106 (6 mice) and 107 CFU (6 mice) S. pneumoniae per mouse. Comparison of the clinical scores of pre-symptomatic and symptomatic periods was made using a Kruskal- Wallis one-way ANOVA

**Figure 2:**
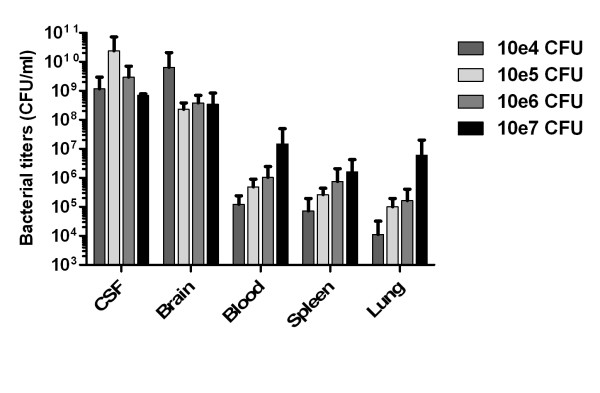
**Bacterial outgrowth**. Bacterial titers in CSF, brain (central nervous system compartment), and blood, spleen, and lung (systemic compartment) at the end-stage of disease after inoculation with 10^4^, 10^5^, 10^6 ^and 10^7 ^CFU *S. pneumoniae *per mouse. Titers are expressed mean CFU/ml +/- S.E.M.

Bacterial meningitis was confirmed in all 23 mice in the survival study by way of culture of CSF and brain homogenate following death or sacrifice. The average pneumococcal concentration in the CSF and brain homogenates was 2.0 × 10^9 ^CFU/ml and 7.9 × 10^8 ^CFU/ml respectively. Bacterial titers in de CNS compartment (CSF and brain) did not increase with higher inoculation doses (Figure 2). In comparison with the CNS compartment, in the systemic compartment (blood, spleen, lung) bacterial concentrations were much lower (means 1.0 × 10^6^, 4.0 × 10^5 ^and 2.0 × 10^5 ^CFU/ml, respectively), and an increasing bacterial titer was observed with each successive 10-fold increase of bacterial inoculum concentration.

**Figure 3:**
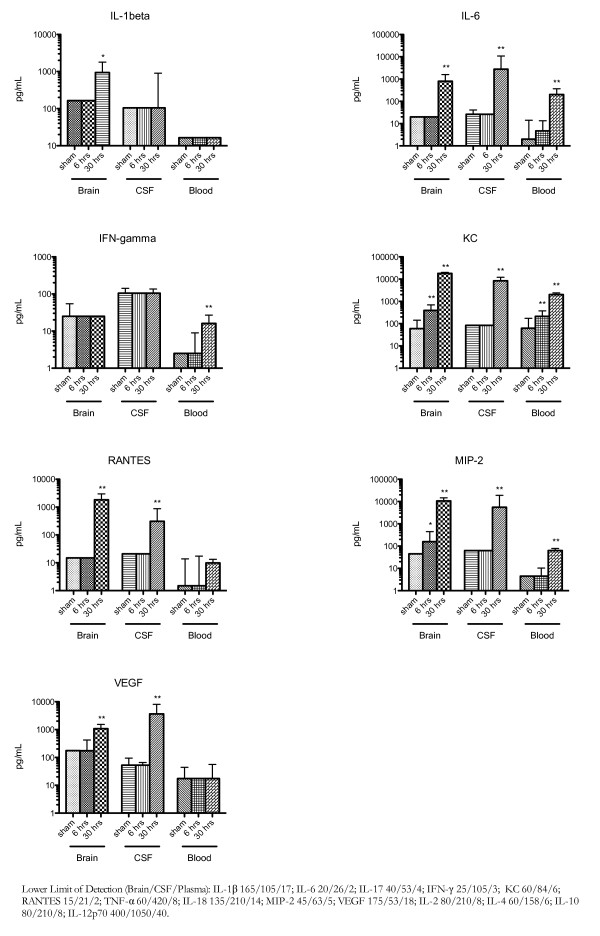
**Cytokine levels in plasma, brain homogenates and CSF**. Median concentrations (expressed in pg/ml) of cytokines in mice inoculated with either NaCl or 10^4 ^CFU/mouse of *S. pneumoniae *and sacrificed after 30 hours, or 6 and 30 hours respectively. Comparisons of cytokine levels between groups were calculated using the Mann-Whitney U test. (*P < 0.05; **P < 0.01).

Mice with pneumococcal meningitis showed increased plasma levels of KC at 6 hours (Figure 3; median 62 versus 213 pg/ml, P = 0.004) and 30 hours (median 62 versus 2031 pg/ml, P < 0.0001) compared to saline inoculated mice. Furthermore, at 30 hours IL-6 (median 2 versus 202 pg/ml, P < 0.001), MIP-2 (median 5 versus 63 pg/ml, P = 0.002) and IFN-γ (median 3 versus 16 pg/ml, P = 0.002) were elevated in plasma of *S. pneumoniae *compared to sham inoculated mice. IL-1β, IL-2, IL-4, IL-10, IL-12p70, IL-17, RANTES, TNF-α, IL-18 and IL-33 were not significantly altered in the plasma of mice with pneumococcal meningitis compared to sham controls.

In brain homogenates, mice with pneumococcal meningitis compared to saline inoculated mice showed elevated levels of KC and MIP-2 at both 6 hours (Figure 3; KC median 60 versus 393 pg/ml, P < 0.0001; MIP-2 median 45 versus 159 pg/ml, P = 0.003) and 30 hours (KC median 60 versus 18116 pg/ml, P < 0.0001; MIP-2 median 45 versus 10637 pg/ml, P < 0.0001) time points. IL-6 (median 20 versus 795 pg/ml, P < 0.0001), IL-1β (median 165 versus 939 pg/ml, P = 0.014), and RANTES (median 15 versus 1823 pg/ml, P < 0.0001) were increased at 30 hours post infection in mice inoculated with pneumococcal meningitis as compared to saline inoculated mice. IL-2, IL-4, IL-10, IL-12p70, IL-17, IFN-γ, TNF-α, IL-18 and IL-33 were not altered in brain homogenates of mice with pneumococcal meningitis compared to sham controls. In CSF of mice with pneumococcal meningitis compared to saline inoculated mice, IL-6 (median 26 versus 2772 pg/ml, P = < 0.001), KC (median 84 versus 8369 pg/ml, P = 0.002), MIP-2 (median 63 versus 5542 pg/ml, P = 0.002) and RANTES (median 21 versus 309 pg/ml, P = 0.005) are elevated 30 hours post infection.

**Figure 4:**
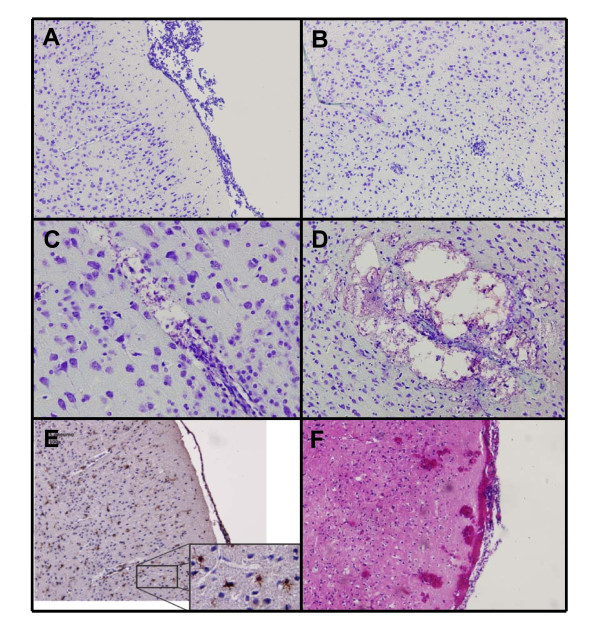
**Brain pathology of mice with pneumococcal meningitis**. Nissl staining of the cortex of mice infected with *S. pneumoniae*, showing extensive leptomeningeal inflammatory infiltrate (A; 100× magnification), perivascular lymphocytic cuffing (B; 100× magnification), perivascular lymphocytic infiltration combined with bacterial overgrowth (C; 200× magnification) and further bacterial overgrowth combined with perivascular necrosis (D; 100× magnification). Iba-1 immunohistochemistry revealed microglial activation (E; 100× magnification). Hematoxylin and eosin staining showed subarachnoidal and cortical hemorrhages (F; 100× magnification).

**Figure 5:**
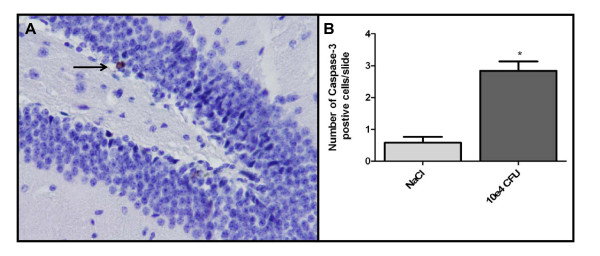
**Neuronal apoptosis**. Caspase-3 immunohistochemistry of 10 μm sections of the middle portion of the hippocampus of the right cerebral hemisphere of mice inoculated with 10^4 ^CFU *S. pneumoniae *at 30 hrs post infection (panel A; 200× magnification). Mice inoculated with *S. pneumoniae *showed significantly more hippocampal apoptosis at 30 hrs post infection than NaCl infected control mice (panel B, expressed in mean number of Caspase-3 positive cells/section +/- S.E.M.; groups were compared using a Student's *t*-test; *P < 0.001).

Histopathology at 6 hours after infection showed high levels of meningeal inflammation in both peripheral and ventricular CSF compartments, but none of the mice had parenchymal lymphocytic infiltration, hemorrhages, microglial activation, or hippocampal apoptosis (Figure 4A). However, 30 hours after inoculation 3 of the 6 mice showed parenchymal lymphocytic infiltration and pockets of bacteria were seen in 2 of 6 mice, located in the perivascular spaces of the penetrating vasculature (Figure 4b). At 30 hours, 5 of 6 mice had one or more parenchymal, mainly cortical, hemorrhages. Three mice demonstrated subarachnoidal hemorrhages. Extensive diffuse microglial activation was observed mice 30 hours after infection and at end stage-stage of disease at all inoculation doses (Figure 4C), although no quantitative analyses were performed. Neuronal apoptosis in the dentate gyrus of the hippocampus was scored independently by two investigators with a kappa of 0.75. A significant increase in hippocampus neuronal apoptosis was observed at 30 hours post infection and was significantly higher than saline infected mice (0.6 vs. 2.8 cells, P < 0.001; Figure 5).

## Discussion

We developed a murine model of pneumococcal meningitis in which the histopathological and inflammatory features as well as observed complications resemble clinical and pathological findings in humans following bacterial meningitis [1,20]. The most important features of this model lie in the possibility of combining a relatively low dose of inoculum and long period of disease progression, allowing for a reproducible setting to examine clinical features as well as sufficient time to develop the histopathological features seen in a human setting.

In previous murine models, pneumococcal meningitis was established by either 1) direct bacterial inoculation into the CNS, which generally very short survival times and thus limited use for the study of inflammation processes, or 2) intranasal or intraperitoneal inoculation routes, which more closely models the longer physiological inflammatory mechanisms [12]. Unfortunately, mice inoculated via the intranasal or intraperitoneal route often died as the result of sepsis or pneumonia, and only 50% actually developed meningitis [17].

In this model the comparison of clinical score progression between mice with different inoculation doses lead to the following conclusions: first, although the pre-symptomatic period was dose dependent (onset of symptoms were later at lower doses of bacterial inoculation), the duration of the symptomatic period was approximately 11.5 hours and similar between groups. This dose-dependent delayed onset provides a model in which direct inoculation in the CNS results in nearly 100% of mice developing meningitis, combined with a prolonged pre-symptomatic period in which various inflammatory mechanisms may be studied. Second, the clinical features contributing to deterioration were largely similar between the 4 different inoculation groups. For example, at the beginning of the symptomatic period (clinical score > 10) the most important contributing factors of clinical deterioration in all four inoculation groups were weight loss, diminished activity and deficits during neurological examination. At the final clinical assessment during the survival experiment, the most important additional factors of clinical deterioration in all 4 groups were the time to turn to upright position and increasing respiratory problems.

TNF-α, IL-1 and IL-6 are considered to be the early response proinflammatory cytokines that are upregulated early in during pneumococcal meningitis [13]. Surprisingly, TNF-α was not elevated at any time-point in our model. Previous animal models demonstrated that TNF-α was mainly increased during the first 6 to 24 hours of the immune response [21,22]. However, human studies show increased CSF levels of TNF-α but only early in the course of the disease [23,24]. This discrepancy may be explained by the lack of measurements that were performed between 6 and 30 hours after infection. IL-1β, which in humans is increased in the first 18 hours of infection [25], was also markedly increased in brain homogenates, but not in blood in our mice 30 hours after infection. The IL-6 concentrations did significantly increase CSF, brain homogenate and plasma 30 hours after infection. This is consistent with other infection models, in which IL-6, a cytokine displaying both pro- and anti-inflammatory properties [26], has been shown to be upregulated early during infection. In previous pneumococcal meningitis models IL-6 was shown to be involved in CSF leukocyte recruitment and possibly in the regulation of blood brain barrier disruption [27]. The anti-inflammatory cytokine IL-10, which has been shown to downregulate TNF-α, IL-6 and KC was not measurably increased at any time point [28].

Of the chemokines, the functional murine IL-8 homologue KC/CXCL1 and MIP-2/CXCL2 were both markedly increased in CSF, brain homogenate and blood at 30 hours after infection. Furthermore, early upregulation of KC and MIP-2 was also observed as early as 6 hours in brain homogenate, but not in plasma, where only KC was significantly increased. In humans, IL-8 has been shown to be elevated in CSF during pneumococcal meningitis [29], yet in a rabbit meningitis model it was systemic IL-8 that appeared to regulate CSF pleiocytosis [30]. MIP-2, which is produced by astrocytes and microglial cells, but also by monocytes and macrophages, has been shown *in vitro *to be a chemoattractant for monocytes and neutrophils recruitment [29].

Brain histopathology in our model resemble the human situation in pneumococcal meningitis, We found meningeal and parenchymal infiltration, (micro)hemorrhages, perivascular lymphocitic cuffing and perivascular bacterial overgrowth, the beginning of abscess formation, microglial activation, and neuronal apoptosis in the dentate hippocampal gyrus. Parenchymal (micro)hemorrhages were frequently observed (83%) and varied in size and location. These results reflect findings in a recent autopsy series in which microhemorrhages were found in 10 of 16 (67%) patients who died of pneumococcal meningitis [10]. In the clinical setting clinical setting only 1-9% of all patients are documented to have intracranial hemorrhagic complications [4], which is likely to be an underestimation of the actual number of hemorrhages as only radiological evidence was included. In our model no cortical necrosis was observed at any time point, including in mice that died in the survival studies. Cerebral infarctions occur in approximately 30% of patients with pneumococcal meningitis [5,20,31], and cortical necrosis has been modeled successfully in several rat and infant mouse meningitis models [18,22]. Possible reasons for the absence of necrosis may lie in the duration from inoculation until sacrifice, the choice of animal, age of the mice used, and antibiotic treatments used in other models. The underlying mechanisms for both ischemic stroke and hemorrhages remain unclear, though human CSF studies have suggested dysregulation of local coagulation cascade, complement activation, and diffuse cerebral intravascular coagulopathy [10,13,32].

The observations of microglial activation at 30 hours after infection reflect *in vitro *findings in which microglial cells are activated after exposure to *S. pneumoniae *[33]. Similarly, the delayed activation of microglial cells supports the results of a previous study in a rabbit model of pneumococcal meningitis in which increased levels of the microglial derived immunomodulatory protein activin A was found at 12 hours after inoculation [34]. Microglia represent a specific subset of cells related to monocytes and dendritic cells and form the initial line of defense of brain parenchyma against damage, injury and infection and become activated in a toll-like receptor dependent fashion upon pneumococcal exposure [35,36]. Upon activation, microglia produce large amounts of proinflammatory cytokines, as well as reactive oxygen and nitrogen intermediates, thereby possibly playing both neuroprotective and neurotoxic roles [13,37-39]. The role of microglia during pneumococcal meningitis is largely unknown at present, but interest has been fueled by the observation that microglial activation *in vitro *is limited by corticosteroids treatment [40], which has become the standard adjuvant therapy in the treatment of bacterial meningitis in many countries [2,41].

Neuronal apoptosis was first observed in the human autopsy studies of patients who died of bacterial meningitis and was situated in the dentate gyrus of the hippocampus [9]. Cognitive impairments and more specifically learning difficulties have been attributed to hippocampal apoptosis which has been modeled in mice, rats and rabbits [18,42,43]. Furthermore, the adjuvant treatment of corticosteroids has been suggested as a possible factor aggravating hippocampal apoptosis and reducing learning capacity [42,44]. The process of apoptosis most likely occurs in an early caspase independent and a late caspase dependent mechanism [45]. In this model we were able to detect the late stage caspase-3 dependent apoptosis at 30 hours post infection, providing an additional outcome parameter for further pathophysiological and therapeutic investigations.

## Conclusions

The value of this mouse model is that it provides an experimental setting of pneumococcal meningitis which is highly reproducible, and provides several of the most valuable outcome parameters such as bacterial titers, meningeal and parenchymal infiltration, cytokine profiles, microglial activation, neuronal apoptosis in the hippocampus, perivascular infiltration and (micro) hemorrhages. We feel that the integration of these pathological features, which are characteristic of what is observed in human autopsy studies into a single model, is a valuable tool in the further investigation of both pathophysiological and therapeutic intervention studies.

## Competing interests

The authors declare that they have no competing interests.

## Authors' contributions

BM-K and MG equally participated in the planning and conducting of all the herein mentioned experiments, as well as the writing of the manuscript. DT aided in the histological analyses. TP and DB conceived of the study, participated in design and execution and evaluation of the various experiments. DB provided funding and aided in the drafting of this manuscript. All authors read and approved the final manuscript.

## Acknowledgements

This study has been funded by grants from the European Research Council (ERC Starting Grant [Proposal/Contract no. 281156] to D. van de Beek), Netherlands Organization for Health Research and Development (ZonMw; NWO-Veni grant 2006 [Proposal/Contract no. 916.76.023], NWO-Vidi grant 2010 [Proposal/Contract no. 016.116.358] to D. van de Beek), the Academic Medical Center (AMC Fellowship 2008 to D. van de Beek).

## Pre-publication history

The pre-publication history for this paper can be accessed here:

http://www.biomedcentral.com/1471-2334/12/71/prepub

## References

[B1] van de Beek D de Gans J Spanjaard L Weisfelt M Reitsma JB Vermeulen M Clinical features and prognostic factors in adults with bacterial meningitis N Engl J Med 2004 351 18 1849 1859 10.1056/NEJMoa040845 15509818

[B2] van de Beek D de Gans J Tunkel AR Wijdicks EF Community-acquired bacterial meningitis in adults N Engl J Med 2006 354 1 44 53 10.1056/NEJMra052116 16394301

[B3] Brouwer MC Tunkel AR van de Beek D Epidemiology, diagnosis, and antimicrobial treatment of acute bacterial meningitis Clin Microbiol Rev 2010 23 3 467 492 10.1128/CMR.00070-09 20610819 PMC2901656

[B4] Weisfelt M de Gans J van der Poll T van de Beek D Pneumococcal meningitis in adults: new approaches to management and prevention Lancet Neurol 2006 5 4 332 342 10.1016/S1474-4422(06)70409-4 16545750

[B5] Weisfelt M van de Beek D Spanjaard L Reitsma JB de Gans J Clinical features, complications, and outcome in adults with pneumococcal meningitis: a prospective case series Lancet Neurol 2006 5 2 123 129 10.1016/S1474-4422(05)70288-X 16426988

[B6] Brouwer MC Heckenberg SG de Gans J Spanjaard L Reitsma JB van de Beek D Nationwide implementation of adjunctive dexamethasone therapy for pneumococcal meningitis Neurology 2010 75 1533 1539 10.1212/WNL.0b013e3181f96297 20881273

[B7] van de Beek D Schmand B de Gans J Weisfelt M Vaessen H Dankert J Vermeulen M Cognitive impairment in adults with good recovery after bacterial meningitis J Infect Dis 2002 186 7 1047 1052 10.1086/344229 12232850

[B8] Hoogman M van de Beek D Weisfelt M de Gans J Schmand B Cognitive outcome in adults after bacterial meningitis J Neurol Neurosurg Psychiatry 2007 78 10 1092 1096 10.1136/jnnp.2006.110023 17353256 PMC2117539

[B9] Nau R Soto A Brück W Apoptosis of neurons in the dentate gyrus in humans suffering from bacterial meningitis J Neuropathol Exp Neurol 1999 58 3 265 274 10.1097/00005072-199903000-00006 10197818

[B10] Vergouwen MD Schut ES Troost D van de Beek D Diffuse cerebral intravascular coagulation and cerebral infarction in pneumococcal meningitis Neurocrit Care 2010 13 217 227 10.1007/s12028-010-9387-5 20526697

[B11] Schut ES Brouwer MC de Gans J Florquin S Troost D van de Beek D Delayed cerebral thrombosis after initial good recovery from pneumococcal meningitis Neurology 2009 73 23 1988 1995 10.1212/WNL.0b013e3181c55d2e 19890068

[B12] Chiavolini D Pozzi G Ricci S Animal Models of Streptococcus pneumoniae Disease Clin Microbiol Rev 2008 21 4 666 685 10.1128/CMR.00012-08 18854486 PMC2570153

[B13] Mook-Kanamori BB Geldhoff M van der Poll T van de Beek D Pathogenesis and pathophysiology of pneumococcal meningitis Clin Microbiol Rev 2011 24 3 557 591 10.1128/CMR.00008-11 21734248 PMC3131058

[B14] Gerber J Raivich G Wellmer A Noeske C Kunst T Werner A Brück W Nau R A mouse model of Streptococcus pneumoniae meningitis mimicking several features of human disease Acta Neuropathol 2001 101 5 499 508 11484822 10.1007/s004010000326

[B15] Quin LR Moore QC McDaniel LS Pneumolysin, PspA, and PspC contribute to pneumococcal evasion of early innate immune responses during bacteremia in mice Infect Immun 2007 75 4 2067 2070 10.1128/IAI.01727-06 17220305 PMC1865685

[B16] Tan TQ Smith CW Hawkins EP Mason EO Kaplan SL Hematogenous bacterial meningitis in an intercellular adhesion molecule-1-deficient infant mouse model J Infect Dis 1995 171 2 342 349 10.1093/infdis/171.2.342 7844370

[B17] Zwijnenburg PJ Van Der Poll T Florquin S van Deventer SJ Roord JJ van Furth AM Experimental pneumococcal meningitis in mice: a model of intranasal infection J Infect Dis 2001 183 7 1143 1146 10.1086/319271 11237845

[B18] Grandgirard D Steiner O Täuber MG Leib SL An infant mouse model of brain damage in pneumococcal meningitis Acta Neuropathol 2007 114 6 609 617 10.1007/s00401-007-0304-8 17938941

[B19] Koedel U Paul R Winkler F Kastenbauer S Huang PL Pfister HW Lack of endothelial nitric oxide synthase aggravates murine pneumococcal meningitis J Neuropathol Exp Neurol 60 11 1041 1050 11706934 10.1093/jnen/60.11.1041

[B20] Kastenbauer S Pfister H-W Pneumococcal meningitis in adults: spectrum of complications and prognostic factors in a series of 87 cases Brain 2003 126 Pt 5 1015 1025 12690042 10.1093/brain/awg113

[B21] Barichello T dos Santos I Savi GD Florentino AF Silvestre C Comim CM Feier G Sachs D Teixeira MM Teixeira AL Tumor necrosis factor alpha (TNF-alpha) levels in the brain and cerebrospinal fluid after meningitis induced by Streptococcus pneumoniae Neurosci Lett 2009 467 3 217 219 10.1016/j.neulet.2009.10.039 19835931

[B22] Ostergaard C Brandt C Konradsen HB Samuelsson S Differences in survival, brain damage, and cerebrospinal fluid cytokine kinetics due to meningitis caused by 3 different Streptococcus pneumoniae serotypes: evaluation in humans and in 2 experimental models J Infect Dis 2004 190 7 1212 1220 10.1086/423852 15346330

[B23] Brivet FG Jacobs FM Megarbane B Cerebral output of cytokines in patients with pneumococcal meningitis Crit Care Med 2005 33 11 2721 2722 author reply 2722-2723 10.1097/01.CCM.0000187092.73841.82 16276227

[B24] Glimaker M Kragsbjerg P Forsgren M Olcen P Tumor necrosis factor-alpha (TNF alpha) in cerebrospinal fluid from patients with meningitis of different etiologies: high levels of TNF alpha indicate bacterial meningitis J Infect Dis 1993 167 4 882 889 10.1093/infdis/167.4.882 8450254

[B25] Schmidt H Stuertz K Bruck W Chen V Stringaris AK Fischer FR Nau R Intravenous granulocyte colony-stimulating factor increases the release of tumour necrosis factor and interleukin-1beta into the cerebrospinal fluid, but does not inhibit the growth of Streptococcus pneumoniae in experimental meningitis Scand J Immunol 1999 49 5 481 486 10.1046/j.1365-3083.1999.00518.x 10320640

[B26] Gadient RA Patterson PH Leukemia inhibitory factor, Interleukin 6, and other cytokines using the GP130 transducing receptor: roles in inflammation and injury Stem Cells 1999 17 3 127 137 10.1002/stem.170127 10342555

[B27] Paul R Koedel U Winkler F Kieseier BC Fontana A Kopf M Hartung HP Pfister HW Lack of IL-6 augments inflammatory response but decreases vascular permeability in bacterial meningitis Brain 2003 126 (Pt 8):1873 1882 10.1093/brain/awg171 12821529

[B28] Zwijnenburg PJG Van Der Poll T Florquin S Roord JJ van Furth AM Interleukin-10 negatively regulates local cytokine and chemokine production but does not influence antibacterial host defense during murine pneumococcal meningitis Infect Immun 2003 71 4 2276 2279 10.1128/IAI.71.4.2276-2279.2003 12654856 PMC152042

[B29] Spanaus KS Nadal D Pfister HW Seebach J Widmer U Frei K Gloor S Fontana A C-X-C and C-C chemokines are expressed in the cerebrospinal fluid in bacterial meningitis and mediate chemotactic activity on peripheral blood-derived polymorphonuclear and mononuclear cells in vitro J Immunol 1997 158 4 1956 1964 9029138

[B30] Ostergaard C Yieng-Kow RV Larsen CG Mukaida N Matsushima K Benfield T Frimodt-Moller N Espersen F Kharazmi A Lundgren JD Treatment with a monocolonal antibody to IL-8 attenuates the pleocytosis in experimental pneumococcal meningitis in rabbits when given intravenously, but not intracisternally Clin Exp Immunol 2000 122 2 207 211 10.1046/j.1365-2249.2000.01357.x 11091276 PMC1905781

[B31] Weststrate W Hijdra A de Gans J Brain infarcts in adults with bacterial meningitis Lancet 1996 347 8998 399 8598723 10.1016/s0140-6736(96)90577-2

[B32] Weisfelt M Determann RM de Gans J van der Ende A Levi M van de Beek D Schultz MJ Procoagulant and fibrinolytic activity in cerebrospinal fluid from adults with bacterial meningitis J Infect 2007 54 6 545 550 10.1016/j.jinf.2006.11.016 17207860

[B33] Liu X Chauhan VS Young AB Marriott I NOD2 mediates inflammatory responses of primary murine glia to Streptococcus pneumoniae Glia 2010 58 7 839 847 20091781 10.1002/glia.20968 PMC2967038

[B34] Michel U Gerber J E O'Connor A Bunkowski S Bruck W Nau R Phillips DJ Increased activin levels in cerebrospinal fluid of rabbits with bacterial meningitis are associated with activation of microglia J Neurochem 2003 86 1 238 245 12807443 10.1046/j.1471-4159.2003.01834.x

[B35] Nau R Bruck W Neuronal injury in bacterial meningitis: mechanisms and implications for therapy Trends Neurosci 2002 25 1 38 45 10.1016/S0166-2236(00)02024-5 11801337

[B36] Ribes S Ebert S Regen T Agarwal A Tauber SC Czesnik D Spreer A Bunkowski S Eiffert H Hanisch U-K Toll-Like Receptor Stimulation Enhances Phagocytosis and Intracellular Killing of Nonencapsulated and Encapsulated Streptococcus pneumoniae by Murine Microglia Infect Immun 2010 78 2 865 871 10.1128/IAI.01110-09 19933834 PMC2812218

[B37] Colton CA Snell J Chernyshev O Gilbert DL Induction of superoxide anion and nitric oxide production in cultured microglia Ann N Y Acad Sci 1994 738 54 63 7530427 10.1111/j.1749-6632.1994.tb21789.x

[B38] Iliev AI Stringaris AK Nau R Neumann H Neuronal injury mediated via stimulation of microglial toll-like receptor-9 (TLR9) FASEB J 2004 18 2 412 414 14688201 10.1096/fj.03-0670fje

[B39] Marques CP Cheeran MC Palmquist JM Hu S Lokensgard JR Microglia are the major cellular source of inducible nitric oxide synthase during experimental herpes encephalitis J Neurovirol 2008 14 3 229 238 10.1080/13550280802093927 18569457 PMC2583058

[B40] Hinkerohe D Smikalla D Schoebel A Dexamethasone prevents LPS-induced microglial activation and astroglial impairment in an experimental bacterial meningitis co-culture model Brain Res 2010 1329 45 54 20230803 10.1016/j.brainres.2010.03.012

[B41] Tunkel AR Hartman BJ Kaplan SL Kaufman BA Roos KL Scheld WM Whitley RJ Practice guidelines for the management of bacterial meningitis Clin Infect Dis 2004 39 9 1267 1284 10.1086/425368 15494903

[B42] Leib SL Heimgartner C Bifrare Y-D Loeffler JM Täauber MG Dexamethasone aggravates hippocampal apoptosis and learning deficiency in pneumococcal meningitis in infant rats Pediatr Res 2003 54 3 353 357 10.1203/01.PDR.0000079185.67878.72 12788989

[B43] Braun JS Novak R Herzog KH Bodner SM Cleveland JL Tuomanen EI Neuroprotection by a caspase inhibitor in acute bacterial meningitis Nat Med 1999 5 3 298 302 10.1038/6514 10086385

[B44] Zysk G Bruck W Gerber J Bruck Y Prange HW Nau R Anti-inflammatory treatment influences neuronal apoptotic cell death in the dentate gyrus in experimental pneumococcal meningitis J Neuropathol Exp Neurol 1996 55 6 722 728 10.1097/00005072-199606000-00006 8642398

[B45] Mitchell L Smith SH Braun JS Herzog K-H Weber JR Tuomanen EI Dual phases of apoptosis in pneumococcal meningitis J Infect Dis 2004 190 11 2039 2046 10.1086/425520 15529270

